# Teaching human parasitology in China

**DOI:** 10.1186/1756-3305-5-77

**Published:** 2012-04-20

**Authors:** Guanghui Zhao, Shenyi He, Lin Chen, Na Shi, Yang Bai, Xing-Quan Zhu

**Affiliations:** 1 Department of Parasitology, Shandong University School of Medicine, Jinan, Shandong Province, 250012, P R China; 2 State Key Laboratory of Veterinary Etiological Biology, Key Laboratory of Veterinary Parasitology of Gansu Province, Lanzhou Veterinary Research Institute, Chinese Academy of Agricultural Sciences, Lanzhou, Gansu Province, 730046, P R China; 3 College of Animal Science and Technology, Yunnan Agricultural University, Kunming, Yunnan Province, 650201, P R China

**Keywords:** Human Parasitology, Teaching, Education, Reform, China

## Abstract

China has approximately one-fifth of the world’s population. Despite the recent success in controlling major parasitic diseases, parasitic diseases remain a significant human health problem in China. Hence, the discipline of human parasitology is considered as a core subject for undergraduate and postgraduate students of the medical sciences. We consider the teaching of human parasitology to be fundamental to the training of medical students, to the continued research on parasitic diseases, and to the prevention and control of human parasitic diseases. Here, we have summarized the distribution of educational institutions in China, particularly those that teach parasitology. In addition, we have described some existing parasitology courses in detail as well as the teaching methods used for different types of medical students. Finally, we have discussed the current problems in and reforms to human parasitology education. Our study indicates that 304 regular higher education institutions in China offer medical or related education. More than 70 universities have an independent department of parasitology that offers approximately 10 different parasitology courses. In addition, six universities in China have established excellence-building courses in human parasitology.

## Review

### Background

Parasitic infections are among the most common communicable diseases of humans in the world [1]. Some of the human parasitic diseases are also listed as the main neglected tropical diseases (NTD), causing death of humans and significant socioeconomic problems [2]. In China, the prevalences of parasitic diseases were 62%–63% in the first national survey of major human parasitic diseases carried out between 1988 and 1992. A second national survey of major human parasitic diseases in China was completed in 2004 and indicated that the prevalences of parasitic diseases had reduced to 22.18% [3].

Parasites also infect animals, and such infections can result in great economic losses. For example, *Toxoplasma gondii* infection is prevalent in a range of animals in China [4-6]. Significant achievements have been made in the prevention and control of some parasitic diseases, with many of the previously widespread and unrestrained diseases now well under control. Moreover, some have been effectively eradicated. Such successes are a result of continued research aimed at revealing the pathogenic mechanisms of the diseases and exploring effective prevention and control methods.

However, it should be recognized that the current state of parasitic infection in humans in some regions remains severe. In some areas, there has been no fundamental change from traditional patterns of prevention and control of parasitic diseases. Meanwhile, new animal-borne parasites and opportunistic pathogenic parasites have emerged [3,7]. Thus, researchers are facing difficulties related to the classification of parasites, including their pathogenic mechanisms, and diagnoses, and especially, the use of preventive and therapeutic drugs and vaccines. To address these difficulties, scientists must undertake unremitting research with the intention to strengthen the delivery of parasitology education and cultivate specialized skills in the field. Unfortunately, parasitology education may not always receive adequate attention in many countries, including China.

Although China has achieved huge success in the control and prevention of some parasitic diseases, current trends of human parasitic disease remain very serious [8]. For example, the number of people in China infected with soil-transmitted nematodes is still more than 100 million [9]; meanwhile, the prevalence of food-transmitted nematodes has increased significantly in recent years, with the number recently reaching 10 million [10]. Traditional parasitic diseases such as malaria and schistosomiasis still occur occasionally in China and, at times, there may be small outbreaks of these diseases. The prevalence and disease occurrence given above show that China should improve its approach to the prevention and control of parasitic diseases. For this, education must be improved; however, the status of human parasitology teaching in China was not exposed internationally. Therefore, we undertook a thorough investigation into the provision of parasitology teaching in China.

For this research, we reviewed the literature related to parasitology education, both from China and other countries, and we examined more than 1800 Chinese college websites to evaluate their parasitology education content. For universities whose websites provided no relevant information, we emailed or telephoned the managers of human parasitology courses to determine their approach to parasitology education. Finally, we summarized the present status of parasitology education in China and listed the number of relevant universities in each province of China.

## Universities providing education in parasitology

According to current official statistics for China [11], there are 2305 regular higher education institutions (HEIs) in China, including 789 universities (48 private universities) and 1246 non-university tertiary institutions (302 private universities). Of the HEIs in China, 307 offer medical or nursing education, including approximately 80 comprehensive universities, 76 special medical education institutions (including medical universities, traditional Chinese medicine universities, pharmaceutical universities, and military medical universities), and 151 non-university tertiary institutions (Table 1).

**Table 1:** Distribution of medical higher education institutions (HEIs) in the provinces of mainland China

**Province**	**Universities**		**Non-university tertiary institutions**
	**Comprehensive universities**	**Specified medical education institutions**	
Beijing	2 (1)	3 (2)	None
Tianjin	1 (1)	2 (1)	1
Hebei	3 (2)	2 (1)	8
Shanxi	1	3 (2)	3
Inner Mongolia	2 (1)	1 (1)	8
Liaojing	2 (1)	5 (1)	3
Jilin	3 (2)	2 (1)	3
Heilongjiang	1 (1)	4 (1)	4
Shanghai	3 (3)	2	8
Jiangsu	6 (4)	3 (1)	6
Zhejiang	7 (2)	2 (1)	4
Anhui*	1	4 (4)	12
Fujian	2 (1)	2 (1)	5
Jiangxi	6 (1)	2 (1)	3
Shandong*	4 (2)	6 (3)	14
Henan	5 (2)	2 (1)	8
Hubei	9 (5)	2	9
Hunan	4 (2)	2 (1)	11
Guangdong*	5 (3)	5 (3)	5
Guangxi*	None	4 (4)	2
Hainan*	None	1 (1)	None
Chongqing*	None	2 (1)	2
Sichuan*	3 (1)	4 (2)	4
Guizhou*	None	3 (3)	8
Yunnan*	2 (1)	2 (2)	6
Tibet	2	None	None
Shanxi	2 (2)	3	9
Gansu*	2 (1)	1	2
Qinghai	1 (1)		1
Ningxia	None	1 (1)	None
Xinjiang	1 (1)	1	2
Total	80	76	151

* Provinces in which parasitic diseases have an epidemic status

The number of universities with an independent parasitology department is given in parentheses.

In 1997, the Degree Committees of the State Department and the State Education Committee integrated medical microbiology and human parasitology to form a new education major called ‘Pathogen Biology’. Some universities also included immunology in this integrated program to form a comprehensive discipline called ‘Immunology and Pathogen Biology’. At present, there is no independent parasitology discipline in HEIs in China. HEIs that set up independent medical microbiology and human parasitology major programs in the past have now integrated them. However, at some higher-level universities in China (more than 70 universities), the two majors have been formally unified, but parasitology remains as an independent department (as either Department of Human Parasitology or Department of Medical Parasitology). These universities are among the best medical education institutions in China, and even though they did not set up independent parasitology departments, education and research in parasitology have been given a high degree of independence.

The data in Table 1 indicate that the distribution of medical HEIs among provinces of mainland China is not equal. The data suggest that the development of medical education has more support in economically well-developed provinces than in economically underdeveloped provinces. However, many of the areas in which parasitic diseases have an epidemic status are economically underdeveloped provinces. This incompatibility is unfavorable for the effective control of parasitic diseases, a situation that should receive the attention of the concerned authorities.

## Parasitology courses

### Human parasitology

Human parasitology, also called medical parasitology, along with microbiology, belongs to the ‘pathogen biology’ educational category. Human parasitology is a medical foundation course and the backbone of parasitology education in China. Virtually all higher-level universities offer this course independently, including most comprehensive universities that provide medical education courses and almost all of the specified medical education institutions. The course’s teaching objective is appropriate for all comprehensive courses of study in medicine (medical majors), including clinical, dentistry, forensic, emergency, laboratory, preventive, and radiation medicine, traditional Chinese medicine and ethno-medicine, as well as anesthesiology and obstetrics and gynecology. The level of education provided by different HEIs and their required medical majors are different; moreover, this course is compulsory at some HEIs but is elective at others. In addition, the teaching semester in which students begin this course and the required class hours are also different. For example, among the eight universities we examined more closely, class requirements are markedly different [12-19] (Table 2).

**Table 2:** Class hour requirements for human parasitology courses at some universities

**University**	**Duration of** **major program**^**a**^	**Total class hours**	
		**Lecture**	**Practice**
Shandong University	5 years	32	32
	6 years 7 years	32 36	32 36
	Foreign students	32	32
Jilin University	5 years 7 years	26 28	20 22
Yanbian University	5 years	26	20
Zunyi Medical College	5 years 4 years	30 16	20 8
Dalin Medical Universities	5 years	24	16
Henan University	5 years	36	18
Beijing University of Chinese Medicine	7 years	18	9
Capital Medical University	5 years 7 years	18 18	5 5

^a^ Four years, a bachelors degree program (nursing specialties); five years, a bachelor degree program (includes clinical, preventive, inspection, and dentistry as well as nursing specialties); six years, a bachelor degree program (Special Medical English Class); seven years, a bachelor, master, or other postgraduate degree program (including clinical, preventive, inspection and dentistry).

Human parasitology has its bases in biology, physiology, biochemistry, human anatomy, embryology, immunology and pathology. It mainly studies the morphology, life cycle, and pathogenesis of parasites, often with an aim to reveal the basic mechanisms related to the prevalence and control or eradication of a parasitic disease or to clarify the relationships between parasites and humans or the external environment.

The required content of the human parasitology course includes a general introduction, followed by information on medical protozoology, medical helminthology, and medical arthropodology. Examples used within the course are mainly related to the common parasites present in China [20-25]. The course’s main content and its teaching format are similar among universities in China; therefore, as an example, we discuss Shandong University’s human parasitology course [12]. This course has two components: lectures and practice. The course’s total class hours are 72, with 36 h of lectures and 36 h of practice (Table 3). This course is taught in English for the university’s five-year clinical major programs (including clinical, preventive, inspection, and dentistry along with nursing specialties). For other major programs, such as those for foreign students, those for six- and seven-year students (i.e., bachelor or master degree programs), and those for eight-year students (i.e., bachelor, master, or other postgraduate degree programs), the human parasitology course is taught in English.

**Table 3:** Human parasitology course class hours by topic in both the lecture and practice portions of the course in Shandong University School of Medicine, China

**Chapter**	**Class period (hours)**	
	**Lecture Practice**	
General introduction	2	2
Medical protozoology	10	
Introduction to medical protozoology	0.5	
*Lobosea* (amoebae)	2	
Flagellates		
*Leishmania donovani*	1	2
*Giardia lamblia*	1	2
*Trichomonas vaginalis*	1	2
Sporozoa		
*Plasmodium*	2.5	2
*Toxoplasma gondii*	1	2
*Cryptosporidium*		
*Pneumocystis carinii*		
Medical helminths		
Introduction to trematodes		
*Clonorchis sinensis*	1.5	2
*Fasciolopsis buski*	0.5	2
*Paragonimus westermani*		
*Pagumogonimus skrjabini*		
*Schistosoma*	2	2
Cestodes (tapeworms)		
Introduction to cestodes		
*Spirometra mansoni*		
*Taenia solium*	1.5	2
*Taenia saginata*		
*Hymenolepis nana*		
*Echinococcus granulosus*	1	2
Nematodes		
Introduction to nematodes		
*Ascaris lumbricoides* (round worm)	1	2
*Trichuris trichiura* (whipworm)		
*Enterobius vermicularis* (pinworm)	0.5	2
*Ancylostoma duodenale* and *Nector americanus* (hookworms)		
*Trichinella spiralis*	0.5	2
*Wuchereria bancrofti* and *Brugia malayi* (Filaria)		
Medical Arthropodology		
Introduction to medical arthopodology		
*Arachnida*	2.5	2
Insecta	5	
Mosquitoes	2	2
Flies, Sandflies	2	2
Fleas	0.5	2
Lice	0.5	
Total	72	36

During the lecturing of the theory portion of the course, multimedia teaching methods are used, with a little additional classroom blackboard writing. The multimedia courseware contains a large number of photographic images of parasites and parasite-affected patients, thus providing vivid and clear descriptions. The evaluation in the theoretical teaching portion is a written examination that forms 80% of the course’s total grade. Examination content is distributed as follows: requiring students to master a topic constitutes 75%–80% of the examination’s total score, requiring students to be familiar with a topic forms 10%–15%, requiring students to know about a topic and self-study makes up 5%, and student participation in seminars constitutes 3%–5%. The examination questions include sections on identification, with multiple-choice questions and questions requiring blanks to be filled with answers, as well as sections on clinical case analysis and others requiring true-false judgment. In addition, educators also include a portion of questions to be written in English to determine the students’ mastery of English terms.

Practical teaching gives priority to observe specimens and perform self-study; however, most practical teaching includes laboratory demonstrations and experiment-related teaching videos that are designed to expand the available knowledge and provide opportunities that practice may not be able to offer. By observing specimens in the practical portion of the course, students can directly observe the morphological features of select parasites. By observing pathological sections, students have the opportunity to grasp and understand various characteristics of human parasitic diseases. Through the practical teaching portion, students are encouraged to become familiar with commonly used experimental diagnostic technologies related to parasitic infections, to enhance their practical abilities, and to improve their basic skills. Through the completion of class work, students are able to learn biological methods and to increase and strengthen their knowledge of parasites’ morphological features. Through the observation of the various classes of parasites, students can learn the main characteristics of each class and learn how to use a classification key to identify species. Through exposure to practical operations related to immunological and molecular biology technologies, students can become familiar with diagnostic methods and new technologies related to parasitic diseases. Such course material is aimed at enhancing students’ knowledge, broadening their experiences, and cultivating innovation [26].

Within the course, a popular segment is that in which every student examines his/her own blood, feces, and facial secretion specimens and inspects laboratory animals to complete a parasitology index and learn to use relevant technological equipment. At the end of the course, they need to complete a report on an aspect of the practical portion of the course. The practical part introduces typical clinical cases and provides clinical discussions about parasitic disease cases. It focuses on training students in various practical aspects related to parasitology and on learning and improving clinical analysis abilities. The course’s practical test contains questions on specimen observation and laboratory operations and includes the submission of a written report. The practical test constitutes 20% of the course’s total grade.

The practical part of the course is an important link to the theoretical portion of the human parasitology course. Teaching practical aspects and integrating them with theory can enhance a student’s professional knowledge and skills, develop strong innovative thinking, and enhance their analytical and problem-solving abilities. Theoretical and practical teaching allows students to fully grasp the basic theories and technologies of human parasitology, thereby laying a foundation for future participation in diagnosing parasitic diseases and parasite-related clinical and preventive medicine.

## Pathogen biology

This course is an integral part of many medical majors, and it provides a partial source of education in human parasitology. Pathogen biology courses include two main topics: microbiology and human parasitology. Some universities refer to the course as microorganism parasitology. The universities that have established this course are predominantly those that have merged the topics of medical microbiology and human parasitology. With the development of pathogen biology major programs, with its fusion of medical microbiology and human parasitology, we foresee an increase in the number of universities in China that offer this course. In addition to offering this course to students in a major program, some universities are providing this course to students seeking adult education or those enrolled in an on-the-job education program or taking part in short-cycle courses.

This course is designed to enable students to obtain basic theoretical knowledge of pathogen biology, to enhance awareness of the medical profession’s “prevention first” approach, and to lay a foundation for learning clinical and preventive medicine. With the ongoing rapid developments in the biosciences, this course should help students become aware of new fields and new concepts within modern pathogen biology and promote the exploration of new contributions to human medicine from research in modern pathogen biology [27,28].

## Clinical parasitology and parasite examination

This is a professional-level course that combines the basics of laboratory medicine with those of clinical practice. It is a specialized course designed for students of laboratory medicine. The course mainly focuses on the morphology and ecology of parasites and expounds on the relationship between parasite pathogenicity and laboratory diagnosis and on control principles associated with parasitic diseases. Through the study of clinical parasitology and parasite examination practices, students are provided with the basic theory, knowledge, and skills of parasitology, in particular, parasite examination technology. This allows students to use common detection techniques and methods associated with parasitic diseases. The course promotes the correct use of parasite examination techniques, and as a result, the levels of prevention and control of parasitic diseases can be improved. This course, when offered at Dalian Medical University, comprises 62 class hours, 32 of which are provided through lectures and 30 through practical teaching [17,29,30].

## Pathogen biology and immunology

This course is currently the main course form that is used to provide parasitology education at a non-university tertiary level. It contains three sections, namely, microbiology, parasitology, and immunology, and is seen as comprehensive but actually superficial as the content in each section is limited. The content related to parasites in this course is restricted to basic knowledge about the most classic and common parasites. Medical major programs that include this course are numerous and include students of clinical, prevention, and laboratory medicine.

A pathogen biology and immunology course mainly teaches basic theories of medical immunology, pathogenicity, immunity, and diagnosis technology, and provides information on preventive measures specific to medical microbes; morphology, life cycle, and ecological aspects of parasites; and the occurrence of popular parasitic diseases. The course is intended to help clarify both contradictory and consistent relationships between parasites and their environment, to help students master basic medical microbiology, immunology, and parasitology knowledge and skills, and to provide a theoretical basis in effective diagnosis, prevention, and treatment measures for infectious parasitic and infectious diseases [31,32].

## Other parasitology courses

Many HEIs have established specialized parasitology courses for postgraduate students. These include courses entitled Advanced Parasitology [Zhongshan (Sun Yat-sen) University] [33], Modern Pathogen Biology (Fudan University) [34], Advances in Pathogen Biology Research (Shandong University) [12], Parasitic Infection and Immunology (Shantou University) [35], and Diagnosis, Treatment and Prevention of Parasitic Diseases (Sichuan University) [36].

## Excellence-building courses

The Ministry of Education in China began the development of ‘Excellence-building Courses’ in 2003. The project focuses on the innovative power of national colleges and universities and takes at least five years to build approximately 1500 excellence-building courses. Well-qualified teachers use superior teaching methods, materials, and education management practices to teach these excellent courses. A network-based curriculum is established to spread research data and knowledge, provide relevant information, and offer educational tools to HEI and non-HEI students.

Currently, an increasing number of students are benefiting from this modern approach to education [37]. At the time of writing this article, six universities in China have established national-level excellent course of human parasitology (Table 4). In addition, 13 universities have established provincial-level excellent course of human parasitology, and six other universities have established school-level excellent course of human parasitology.

**Table 4:** National-level human parasitology excellence-building courses in China

**Universities**	**Web sites**
Shandong University	http://www.pathobio.sdu.edu.cn/sdjsc/
Central South University	http://netclass.csu.edu.cn/JPKC2007/CSU/29jishengchong/INDEX.HTML
Southern Medical University	http://jpkc.fimmu.com/jsc/
Zhengzhou University	http://cod.zzu.edu.cn/newware/rtjsc/
Huazhong University of Science & Technology	http://202.114.128.246/shenbao/jsc/index.htm
Sun Yat-sen University	http://jpkc.sysu.edu.cn/jishengchong/index.htm

## Teaching human parasitology for postgraduate students

At present, there are about 40 human parasitology-related postgraduate programs in China, and 18 medical schools and institutions in China have been approved to grant doctoral degrees in human parasitology. Both Masters degree and doctorate programs in medicine usually take three years to complete. Both programs include course work and research components. Master of Medicine students are also required to participate in practical classes as demonstrators in partial fulfillment of their degrees [38].

Both master degree and doctorate students registered in a major program in human parasitology are required to undertake research in molecular parasitology, parasite immunology, or traditional parasitology. The research should focus on new diagnostic techniques, molecular-level mechanisms of pathogenesis, drug resistance, antigenic variation, parasite genomics, and parasite proteomics, etc.

## Education of foreign students

In the 1950s, universities in China began to enroll foreign students, including government-supported and non-supported students. Since 2000, medical education of foreign students has increased markedly and, at present, about 39 medical schools in China enroll non-supported foreign students. These students usually come from neighboring or nearby countries, such as Pakistan, Vietnam, Malaysia, India, Indonesia, and Mongolia. By the end of 2009, China had accepted more than 230,000 students from 190 countries and regions.

However, there is no unified teaching program or consistent use of textbooks for the teaching of human parasitology in China for international students. On the basis of the specific course subject, teachers compile their own English language teaching materials in order to provide foreign students with suitable and practical learning material that matches their educational level [39,40]. In 2011, Shandong University Press published an English language textbook on human parasitology that was written by Chinese parasitology experts [41]. Such a book can provide a more consistent source of parasitology educational material to foreign students. This book is currently viewed as an ideal choice for teaching parasitology in English in China [41].

## Teaching reforms

To overcome current difficulties and new challenges in teaching human parasitology and to integrate better parasitology education into contemporary education, experts and scholars in parasitology are actively exploring ways to reform parasitology education in China. One approach for reform is to reduce the number of student contact hours. To meet teaching targets in human parasitology, there is an increasing use of multimedia facilities to reduce the amount of didactic teaching. The use of a variety of such methods has been reported to help enliven a lesson [42-44].

To enhance the ability to keep abreast of current development in science and technology, both at home and abroad, some universities have started teaching subjects (including human parasitology) bilingually (using an English language textbook, but with lectures and examinations being half in Chinese and half in English). One of the objectives of bilingual teaching is to help students master the field’s specialized vocabulary and improve their ability to read professional literature. Typically, the English content of such textbooks is derived from an original English language edition [45,46], but recently, some Chinese parasitologists have independently written textbooks in English [41].

In the teaching of morphology, observation is important. In order to improve the effectiveness of the practical teaching of observation, new modes have been added [47,48]. For example, at some universities, students have an opportunity to study live animal models that are infected by parasites, or examine stool samples to help them master conventional methods of stool examination.

In addition, many parasitology educators are exploring various new teaching methods, for example, participatory teaching methods [49], problem-based learning models [50], and computer-assisted learning [51], in the teaching of human parasitology. Moreover, some educators are merging various fields within the medical humanities with the teaching of human parasitology [52].

## Conclusion

The need of applied parasitology remains paramount, and further development of applied parasitology in China requires a new generation of well-educated parasitologists and other scientists. The biggest challenge in developing this new generation is how to integrate the establishment of high-quality parasitological teaching with a reduced instruction time in human parasitology. We believe that the quality of human parasitology teaching is improving dramatically. Nevertheless, reforms to human parasitology teaching may still be required in order to meet new challenges.

## Competing interests

The authors declare that they have no competing interests.
